# Phosphorylation of the regulatory light chain of myosin in striated muscle: methodological perspectives

**DOI:** 10.1007/s00249-016-1128-z

**Published:** 2016-04-15

**Authors:** Haiyang Yu, Samya Chakravorty, Weihua Song, Michael A. Ferenczi

**Affiliations:** Lee Kong Chian School of Medicine, Nanyang Technological University, Experimental Medicine Building, Level 3, 59 Nanyang Drive, Singapore, 636921 Singapore

**Keywords:** Myosin, Regulatory light chain, Phosphorylation, Muscle contraction, Cardiovascular disease, Mechanochemistry

## Abstract

Phosphorylation of the regulatory light chain (RLC) of myosin modulates cellular functions such as muscle contraction, mitosis, and cytokinesis. Phosphorylation defects are implicated in a number of diseases. Here we focus on striated muscle where changes in RLC phosphorylation relate to diseases such as hypertrophic cardiomyopathy and muscular dystrophy, or age-related changes. RLC phosphorylation in smooth muscle and non-muscle cells are covered briefly where relevant. There is much scientific interest in controlling the phosphorylation levels of RLC in vivo and in vitro in order to understand its physiological function in striated muscles. A summary of available and emerging in vivo and in vitro methods is presented. The physiological role of RLC phosphorylation and novel pathways are discussed to highlight the differences between muscle types and to gain insights into disease processes.

## Introduction

**Fig. 1:**
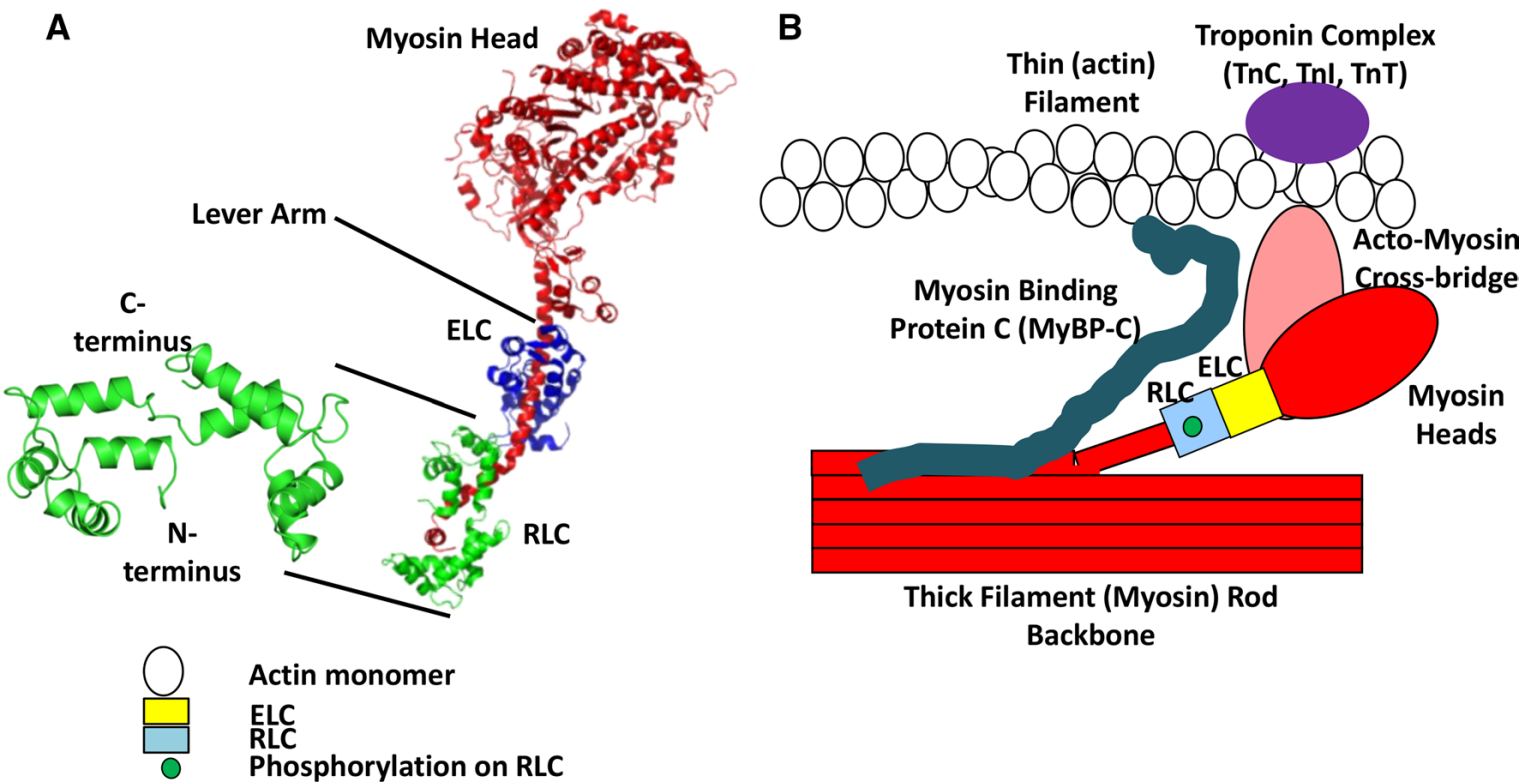
RLC structure. **a** Chicken skeletal muscle isoform of RLC crystal structure with the helix-loop-helix showing four EF hands separated by loops (*left*). The N- and the C-termini are shown (PDB ID # 2MYS; (Rayment et al. 1993) that wrap around the C-terminus side of the chicken skeletal myosin S1 sub-fragment (*right*) (Rayment et al. 1993). The N-terminal amino acids 1-20 are missing from the crystal structure due to its flexibility (inherent disorder). The myosin head is the motor domain of the myosin molecule, the site of actin binding and contains the ATP hydrolysis pocket. The regulatory (RLC) and essential (ELC) light chains wrap around the lever arm conferring rigidity and stability to this long alpha-helical rod. **b** A simplified pictorial representation of the acto-myosin structure with RLC. The diagram shows the thick (myosin containing, *red*) filament backbone and thin (actin containing, *white circles* represent actin monomers) filament with an acto-myosin cross-bridge. The ELC (*yellow box*) and phosphorylated RLC (*blue box*) are in the neck region of the myosin heavy-chain monomer. The myosin head interacts weakly or strongly with the thin filament forming a cross-bridge structure. The Troponin complex (*purple oval*) on the thin filament comprising of troponin C, troponin I, and troponin T activate muscle contraction via calcium binding. Myosin binding protein C (MyBP-C) (*cyan*) is associated with the thick filament backbone and also interacts with the thin filament (not drawn to scale). (Figure is modified from Fig. 6c of Farman et al. 2009)

Muscle contraction is driven by cyclical interaction between the thin filament protein actin and the thick filament protein myosin (Spudich 2001). The myosin family of proteins consists of more than two dozen distinct members (Ferenczi 2000; Pfitzer 2001). Myosin molecules are large (with a size of about 500 kDa), actin-binding ATPases, which act as molecular motors to provide a variety of movement-associated functions (Hartman and Spudich 2012; Spudich 2001; Syamaladevi et al. 2012). The 440-kDa Myosin II dimer, arranged into bipolar filaments, also known as thick filaments and containing 294 molecules (Atkinson and Stewart 1991), are the molecular motors responsible for muscle contraction. Myosin is the mechanochemical energy transducer, whose motor domain (or head) interacts with actin in the thin filament generating force, power, and shortening (Irving et al. 1992). Each myosin molecule is a hexameric protein containing two heavy chains (MHC) and four light chains. The stability of the α-helical neck region of the S1 subfragment of each myosin heavy chain, also known as the lever arm region, is stabilized by two small light-chain proteins known as MYL1 (the alkali light chain, or the essential light chain, ELC, ~25 kDa) and MYL2 (the regulatory light chain or RLC, ~19 kDa) (Fig. 1). Both RLC and ELC have a structure closely related to that of the EF hand calcium binding protein family such as troponin C and calmodulin (Weeds 1969). However, unlike other EF hand proteins, RLC has only one Ca^2+^/Mg^2+^ binding EF hand domain between amino acids 37 and 48 (Collins 1976; Rayment et al. 1993). Thus, modulation of contraction by RLC phosphorylation has attracted much interest.

In striated muscles, activation of the myosin motor is mediated by thin-filament activation upon Ca^2+^ binding (on/off) to the actin–troponin–tropomyosin complex, not by RLC phosphorylation. RLC phosphorylation acts as a modulator and not as a switch in striated muscle (Sweeney et al. 1993; Szczesna 2003). In striated muscle, the RLC phosphorylation state may function as the molecular memory of recent activation and contractile events by altering actomyosin interactions after repeated muscle activation (Kamm and Stull 2011; Matsumura et al. 2001; Scruggs and Solaro 2011). It has been proposed that phosphorylation induces movement of the myosin head away from the thick filament backbone toward the thin filament, thus increasing the probability of myosin attachment to actin (Colson et al. 2010; Levine et al. 1996). Below we describe the structure of RLC, the phosphorylation sites, and their functional significance.

## Structure, isoforms, phosphorylation sites, and function of RLC

### Structure

RLC (~19 kDa) is a member of the EF-hand superfamily (which is a helix-loop-helix structural domain or motif found in a large family of calcium-binding proteins). The N-terminal domain is similar in structure to calmodulin. RLC is non-covalently bound to the C-terminus of myosin S1 subfragment (Fig. 1b). The N-terminal domain of RLC wraps around the region between amino acids Asn 825 and Leu 842 at the C-terminal end of myosin S1. The C-terminal domain of RLC binds in the region between Glu 808 and Val 826 of myosin S1 (Rayment et al. 1993). The number of phosphorylatable serines of RLC differs between species and between tissues indicative of the presence of unique tissue-specific isoforms and phosphovariants, thus providing scope for a variety of functions. Although the isoforms of RLC are similar in structure, in particular in the divalent cation-binding sites and proximal phosphorylatable serines in both smooth and striated muscles, they have relatively low sequence similarity (~57 %), suggesting functional differentiation (Scruggs and Solaro 2011).

### Isoforms

Within the same tissue, there are multiple isoforms of RLC. In adult human skeletal muscle, there are three major RLC isoforms: two fast isoforms, namely type I (NCBI Accession # P24732) and type II (NCBI Accession # P02608), and one slow isoform (NCBI Accession # P10916, same as ventricular RLC), where fast and slow describe the physiological behavior of different muscle groups. These three isoforms are encoded by different genes in the human chromosome 16, but with a high degree of sequence homology (Szczesna 2003).

In human heart, there are two different RLC isoforms expressed from separate genes, namely the atrial isoform (MYL2a) (NCBI Accession # M94547) encoded from a gene in chromosome 7 and the ventricular isoform (MYL2v) (NCBI Accession # P10916) encoded from a gene in chromosome 12, with the ventricular ones expressed in both ventricular myocardium and slow twitch skeletal muscle (MYL2f) (Kubalak et al. 1994; Price et al. 1980; Sarkar et al. 1971; Seidman and Seidman 1998).

### Phosphorylation sites

#### Mammalian skeletal muscle

**Fig. 2:**
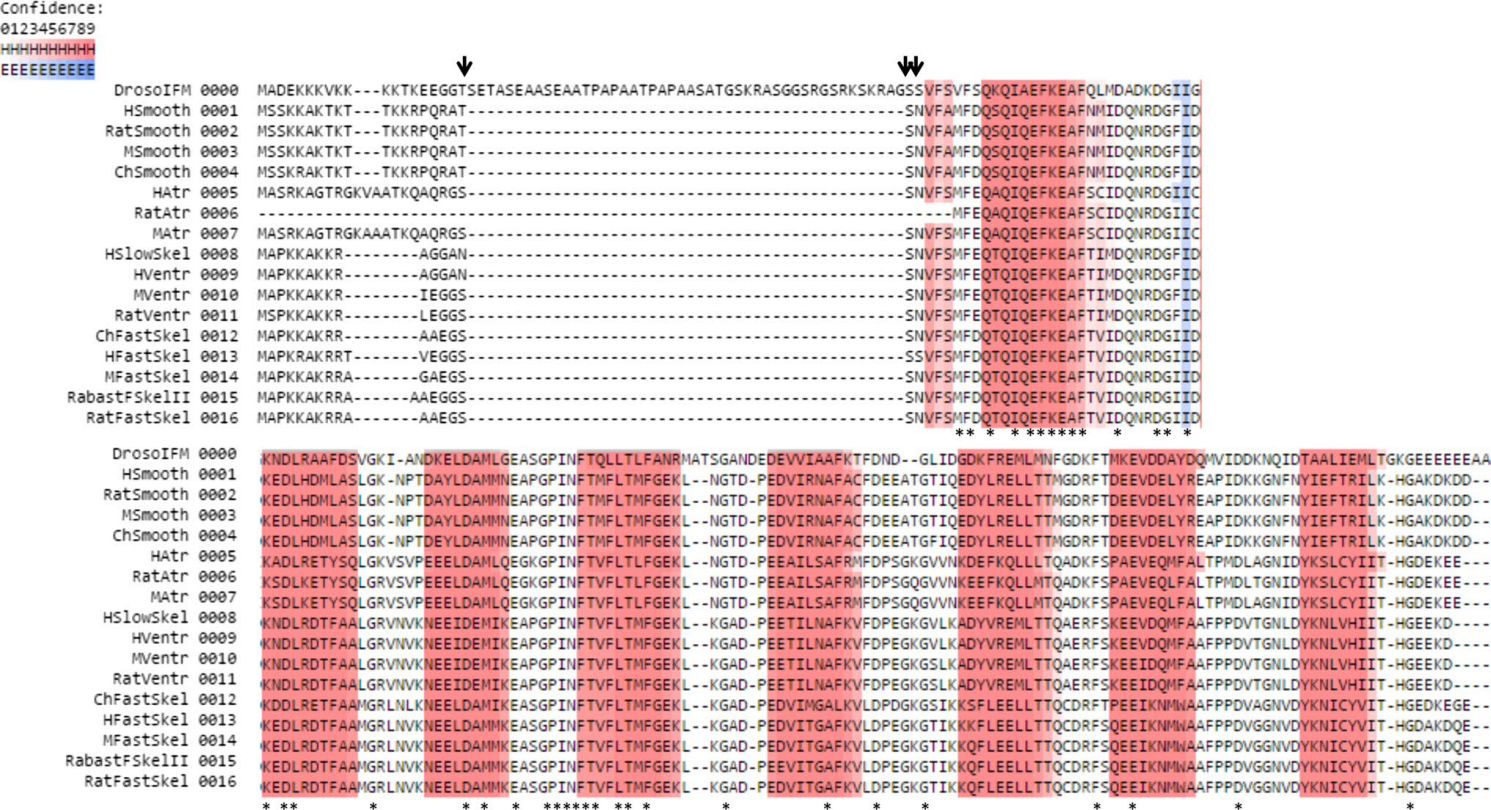
Multiple sequence alignment (MSA) of different RLC isoforms from different laboratory model organisms and humans showing eight conserved predicted helices (*red*) forming part of the conserved four EF-hand secondary structure. The helix-loop prediction was performed in the Ali2D Bioinformatics toolkit of the Max Plank Institute of Developmental Biology (http://toolkit.tuebingen.mpg.de/ali2d). The degree of confidence in prediction secondary structure is color coded in shades of increasing color intensity indicating increasing prediction confidence (for helix (H): *white to dark red*, for loops (E): *white to dark blue*). The *black arrows* indicate the conserved sites of physiologically relevant phosphorylation sites of RLC across species and tissues. The *asterisks* (*) indicate conserved residues across the sequences in the MSA. Sequences were retrieved from NCBI. Abbreviations used for biological sources of RLC sequences with their respective gene names and NCBI protein accession numbers are as follows: DrosoIFM (*Drosophila* indirect flight muscle, *MLC2*, P18432.2), HSmooth (human smooth muscle, *MYL12B*, O14950.2), RatSmooth (rat smooth muscle, *MYL12B*, P18666.3), MSmooth (Mouse smooth muscle, *MYL12B*, Q3THE2.2), ChSmooth (chicken smooth muscle, *MYL12A*, P24032.2), HAtr (human atrial muscle, *MYL7*, Q01449.1), RatAtr (rat atrial muscle, *MYL7*, NP_001099487.1), MAtr (mouse atrial muscle, *MYL7*, Q9QVP4.1), HSlowSkel (human slow skeletal muscle, same as human ventricular), HVentr (human ventricular muscle, *MYL2*, P10916.3), MVentr (mouse ventricular muscle, *MYL2*, P51667.3), RatVentr (rat ventricular muscle, *MYL2*, P08733.2), ChFastSkel (chicken fast skeletal muscle*, MYLPF*, P02609.2), HFastSkel (human fast skeletal muscle, *MYLPF*, Q96A32.1), MFastSkel (mouse fast skeletal muscle, *MYLPF*, P97457.3) RabastFSkelII (rabbit fast skeletal type II muscle, *MYLPF*, P02608.3), RatFastSkel (rat fast skeletal muscle, *MYLPF*, P04466.2)

**Fig. 3:**
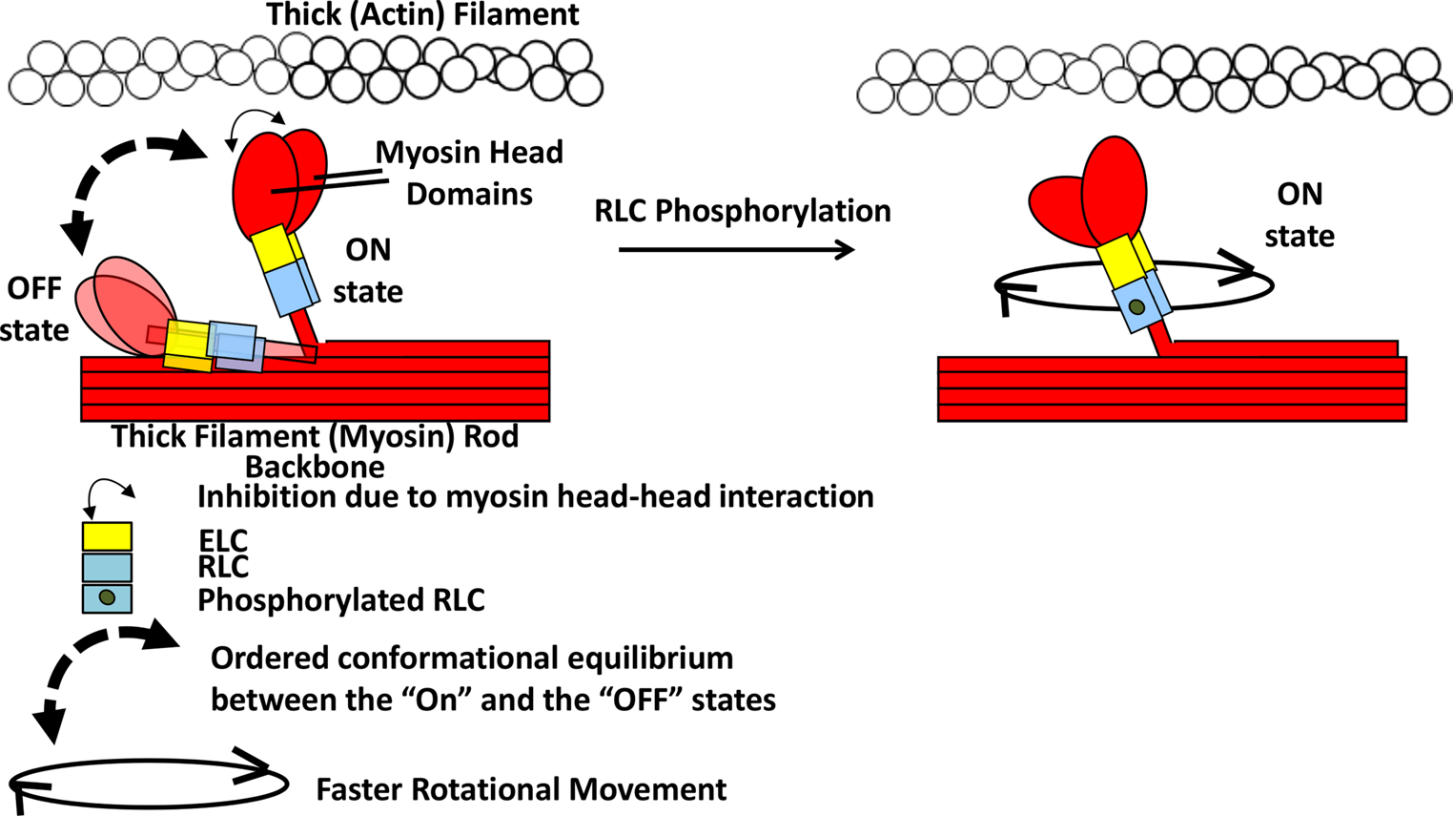
Graphical representation of the effect of RLC phosphorylation on myosin head and lever arm arrangement. (Figure modified from Fig. 6c of Farman et al. 2009) Model based on skeletal muscle (Duggal et al. 2014; Midde et al. 2013) and cardiac muscle (Kampourakis and Irving 2015). In this model, without RLC phosphorylation, the myosin S1 and RLC regions are in conformational equilibrium between lying/binding to the thick filament backbone surface (OFF state) and moving away towards thin filament (ON state) controlled by the RLC and the thick filament surface. This equilibrium is shifted towards the ON state upon RLC phosphorylation, possibly due to weakened electrostatic interaction between the added phosphate and negatively charged thick filament surface. The entire thick filament arrangement of myosin heads and other sarcomeric proteins (MyBP-C, Troponin complex etc.) are not shown for simplicity. Not drawn to scale. (Notes: The ON and OFF states mentioned here are not related to the activation/deactivation with calcium binding/unbinding to Troponin C. The OFF-state myosin heads without RLC phosphorylation is drawn here lying on the thick filament backbone in the same direction as in the ON state, but it is possible that the OFF-state heads may fold on the opposite direction interacting with the thick filament backbone as drawn previously in Fig. 5 of Kampourakis and Irving 2015)

Vertebrate skeletal muscle RLC is phosphorylated at two serine (Ser) residues, Ser14 and Ser15 (Fig. 2), by a skeletal muscle-specific Ca^2+^/calmodulin-dependent myosin light-chain kinase (skMLCK) (Blumenthal and Stull 1980). The specific roles of the two neighboring RLC phosphorylation sites are unknown, but RLC phosphorylation at either site has a profound effect on force potentiation (isometric twitch tension potentiation) in fast, but not in slow, skeletal muscle (Moore and Stull 1984). Tarantula muscle has very thick myosin filaments, and their structure has been well studied by electron microscopy. It has been found that RLC phosphorylation changes the orientation of the myosin head on the thick filament backbone and is the contractile basis of force potentiation in striated muscles. It was found that force potentiation occurs by sequential and cooperative phosphorylation of two RLC sites in tarantula thick filament: first Ser35 by Protein Kinase C (PKC) and then Ser45 by Myosin Light Chain Kinase (MLCK) (Espinoza-Fonseca et al. 2015). This is further validated by molecular dynamic simulations showing that the diphosphorylation of both tarantula smooth and striated muscle stiffens the N-terminal extension of RLC (region where phosphorylation occurs) so as to prevent its docking back towards the myosin S1 region, and in this way helps the free myosin head with Ser35 phosphorylation to remain away from thick filament backbone and available for actin binding (Alamo et al. 2015). It must, however, be borne in mind that tarantula thick filament may function very differently from vertebrate thick filaments. However, electron microscopic analyses of rabbit skeletal muscle showed that RLC phosphorylation has a major role in structural transitions, and modifies the mobility and disorder of the myosin head and myosin head crown/helical arrangement in the thick filament backbone resulting in force potentiation (Levine et al. 1996). RLC phosphorylation-induced force potentiation is enhanced in both disease (muscular dystrophy) and aging, both of which affect neuro-muscular coupling (Smith et al. 2010). In these disorders, RLC phosphorylation may be a compensatory mechanism enhancing force potentiation. These effects and their physiological relevance may stem from RLC phosphorylation-induced movement of myosin heads away from the thick filament backbone towards the thin filament target zones facilitating contraction (see Fig. 3).

#### Mammalian cardiac muscle

In striated cardiac muscle from mouse or rat, RLC exists in three states: unphosphorylated, singly phosphorylated at serine 14 or 15, and doubly phosphorylated at serine 14 and 15. These sites are mostly phosphorylated by a cardiac-specific MLCK (cMLCK), and partly by zipper-interacting kinases (ZIP Kinase). While mouse and rat possesses phosphorylatable sites at both Ser14 and Ser15, human cardiac RLC (cRLC) has only one single phosphorylatable site at Ser15 (Fig. 2) (Lyon et al. 2009; Scruggs et al. 2010). Interestingly, human cRLC contains asparagine (Asn) instead of Ser14. Asn14 can be deamidated to aspartic acid (Pasparakis et al. 2010), chemically mimicking the negatively charged state of a phosphorylated serine, although there is no evidence that Asn14 is indeed deamidated physiologically. Therefore, human cRLC may exist in three distinct charged species in vivo: one unphosphorylated, one singly phosphorylated, and one phosphorylated/deamidated mimicking a doubly phosphorylated state (Scruggs et al. 2010). Moreover, isoform compositions expose differences between species. For example, human cRLC has two distinct charged isovariants with identical molecular weights, with one being more highly expressed and highly phosphorylated (Morano 1992; van Der Velden et al. 2001). Moreover, in non-failing human heart, the α-MHC content comprise as much as 10 % of the total MHC in cardiac sarcomeres (Miyata et al. 2000). This indicates that the isoenzymes in human cardiac muscle are not only created by the two different isovariants of RLC, but different MHC composition of the adult human left ventricle. In contrast, rat has one ventricular RLC isoform but the three isoenzymes are formed as mixtures of α and β MHC (Kumar et al. 1986). Phosphorylation of RLC enhances the affinity of cross-bridges for action, or the rate of cross-bridge entry into the force generating states by moving cross-bridges closer to the thin filament (Colson et al. 2010). Physiologically, RLC phosphorylation may enhance the calcium sensitivity of cardiac myofilaments (Morano et al. 1985). Chris Toepfer sees an effect of phosphorylation in rat cardiac trabeculae at saturating calcium, so the effect cannot be fully attributed to enhancement of calcium sensitivity (Toepfer et al. 2013). Engineered in vivo mutations highlight the role of Ser14 and Ser15 phosphorylation (Ding et al. 2010; Sanbe et al. 1999). Phosphorylation of cRLC at both sites is required for normal cardiac ejection dynamics at baseline and also following β1-adrenergic stimulation. Genetic engineering of the phosphorylation sites showed that Ser15 of cRLC is specifically phosphorylated by cMLCK (Kampourakis and Irving 2015), confirming that Ser15 is a physiologically relevant site.

#### Mammalian smooth muscle

In contrast to striated muscle, smooth muscle is activated by RLC phosphorylation at Ser19 of RLC. Both Thr18 and Ser19 are phosphorylated by smooth muscle specific myosin light chain kinase (smMLCK) in the presence of calcium and calmodulin (Pearson et al. 1984). Smooth muscle activation is, however, more complex than merely being turned on by phosphorylation of RLC and turned off by dephosphorylation. Smooth muscle force generation is maintained over long periods of time even though the extent of RLC phosphorylation declines over time (Driska et al. 1981). In vitro, smooth muscle RLC can be simultaneously phosphorylated at Thr18 position along with Ser19 by integrin-linked kinases and zipper-interacting kinases (ZIPK) (Ihara and MacDonald 2007; Wilson et al. 2005). Di-phosphorylation may occur in diseases of smooth muscle (Walsh 2011). Compared with mono-phosphorylation exclusively at Ser19, di-phosphorylation at both Ser19 and Thr18 slows relaxation of arterial smooth muscle by reducing the rate of dephosphorylation of the RLC (Sutherland and Walsh 2012). Using Mn^2+^-phos-tag gel electrophoresis technology for detecting phosphorylated variants of proteins, Aguilar et al. showed three differentially charged species of RLC in cultured uterine smooth muscle myocyte: unphosphorylated, singly and doubly phosphorylated (Aguilar et al. 2011). In spite of finding di-phosphorylated RLC in smooth muscle, mono-phosphorylated RLC at Ser19 by Ca^2+^/calmodulin-dependent smMLCK predominates physiologically in adult smooth muscle tissues. In vitro, other phosphorylatable sites of RLC in smooth muscle are found. Calcium-activated phospholipid-dependent protein kinase C (PKC) phosphorylates Ser1, Ser2, and Thr9 of smRLC, which reverses the increase in ATPase activity associated with smMLCK phosphorylation at Ser19 (Bengur et al. 1987; Ikebe et al. 1987).

### What do we know about the role of RLC phosphorylation?

#### Skeletal muscle

Unlike in smooth muscle, RLC phosphorylation does not act as an activation switch but modulates striated muscle contraction. However, as in smooth muscle, RLC phosphorylation in skeletal muscle may also contribute to the release of the inhibition due to head–head and head–thick filament backbone interactions, facilitating binding with actin (Craig et al. 1987; Zhao et al. 2009). However, this role is not well understood. RLC phosphorylation enhances actomyosin interactions to accelerate myosin ATPase kinetics and force generation following calcium activation in skeletal muscle (Sweeney and Stull 1990). This is supported by structural studies which show that phosphorylation promotes the movement of the myosin heads away from the thick filament backbone resulting in the rotation of the cross-bridge/lever arm (Fig. 3) (Irving et al. 2000; Ushakov et al. 2011). In skeletal muscle, RLC phosphorylation may affect stiffness of the lever arm under isometric conditions and may be bringing the cross-bridges closer to the thin filament, without an effect on isometric tension generation. However, in non-isometric conditions, in vitro motility assays showed that actin filament gliding slows down upon RLC phosphorylation due to an increase in the myosin duty cycle (Greenberg et al. 2009). However, the structure of the myosin molecule or filament bound to a glass substrate as used in gliding assays may be substantially different from that encountered in muscle, which may result in different effects of phosphorylation in these two types of experiments.

#### Effect of RLC phosphorylation on actomyosin ATPase

Myosin ATPase activity, in the absence of actin, is not affected by RLC phosphorylation (Persechini and Stull 1984). In fibers, phosphorylation of RLC enhances Ca^2+^ sensitivity of force development, but only slightly accelerated ATPase activity in skeletal muscle with low statistical significance in both reconstituted myosins and in permeabilized muscle fibers (Szczesna et al. 2002).

#### Effect of RLC phosphorylation on force development and shortening

Using rabbit psoas muscle fibers, it was found that RLC phosphorylation slowed muscle shortening velocity by ~40 % under physiological fatigue condition (such as 30 °C, low pH, and increased Pi concentration) of muscle (Karatzaferi et al. 2008), independent of myosin and in both relatively lower and higher temperatures. It has been proposed that at higher temperature in fatigued muscle with phosphorylated RLC, the myosin heads bind to the thick filament backbone, forming ordered arrays. This binding results in the myosin heads being further away from the thin filament binding sites upon Ca^2+^ activation, and increasing the fraction of myosin heads in non-force generating states during extreme fatigue conditions. Albeit, it is not known if inhibition of shortening velocity during fatigue is due to the direct role of RLC phosphorylation on nucleotide binding to myosin motor domain or not.

Interestingly, recently it was found that in a skeletal myosin light chain kinase knock-out (skMLCK−/−) mouse, the extensor digitorum longus (EDL) muscle exhibits no post-tetanic force potentiation after potentiating stimuli compared to that in wild-type control mice (Gittings et al. 2016). This observation highlights the link between RLC phosphorylation in force potentiation as without skMLCK, the majority of skRLC would remain non-phosphorylated.

#### Cardiac muscle modulation by RLC phosphorylation

The proportion of cardiomyocytes with well-organized sarcomeric structure increased in vitro in cells containing phosphorylated RLC compared to unphosphorylated cells in rat and zebra fish (Aoki et al. 2000; Seguchi et al. 2007). This indicated that RLC phosphorylation is involved during the development of cardiac sarcomeres. Some studies do not show a role for RLC phosphorylation; for example, it was shown that cardiac performance can be increased without increasing RLC phosphorylation in mice (Chang et al. 2013). Increased performance may be achieved by activating other signaling pathways such as CaMKII and with the help of neuregulin. However, RLC phosphorylation facilitates force production by increasing maximal isometric tension, calcium sensitivity of force, and the rate of force redevelopment at intermediate and saturating calcium activation levels (Olsson et al. 2004; Szczesna et al. 2002). Overall, RLC phosphorylation facilitates actomyosin interaction, most likely accelerates actomyosin kinetics, and enhances power output in cardiac muscle. These functional enhancements may promote sarcomeric organization during development. X-ray diffraction experiments on cardiac trabeculae with in situ RLC phosphorylation suggests a movement of the myosin head towards the thin filament upon phosphorylation (Colson et al. 2010), which might increase the probability of acto-myosin cross-bridge formation (Scruggs and Solaro 2011).

Some of the familial hypertrophic cardiomyopathy (FHC) mutations known in human cardiac RLC are found in the region of the Ser15 phosphorylation site, affect RLC phosphorylation and have a physiological effect (Szczesna et al. 2001). In Szczesna et al.’s report, of particular relevance, was the decrease in RLC phosphorylation in the A13T (site close to Ser15 site) and R58Q (site far from Ser15 site) mutations causing abnormal α-helical content and reduced Ca^2+^-binding properties of RLC, which were compensated upon subsequent phosphorylation (Szczesna et al. 2001, 2002). This indicates that RLC phosphorylation may be a key compensatory mechanism to attenuate the physiological consequences of FHC mutations in the heart. Toepfer et al. found that enrichment of RLC phosphorylation enhances force and power output in rat cardiac trabecular muscle (Toepfer et al. 2013). This study also shows that in both rats and humans, RLC phosphorylation increases with the severity of heart failure. This may support the view that RLC phosphorylation is a compensatory mechanism in cardiac disease. During the power stroke, the cross-bridge converts the torque generated in the myosin monomer into a linear displacement of the filaments with respect to each other (Burghardt and Sikkink 2013). Therefore, the possible immediate effect of RLC phosphorylation could be the movement of the myosin motor to facilitate inter-filament interactions, transient cross-bridge formation and detachment, and muscle contraction. Stelzer et al. incorporated MLCK to increase RLC phosphorylation in a MLCK knock-down background in permeabilized murine myocardium and found increases in the amplitude and rate of force development during stretch, i.e., enhancement of the stretch activation response. This further indicates that RLC phosphorylation increases force and power output in vivo, at least in the murine myocardium, by possibly increasing the rate of acto-myosin cross-bridge recruitment. Further work is required to understand the role of RLC phosphorylation on actomyosin kinetics, on stretch-activation, and on the behavior of the whole heart.

The phosphorylation state of other myofibrillar proteins in both thick and thin filaments, namely that of myosin binding protein C (MyBP-C) and troponin I (TnI), are also affected by in vivo mutations of cRLC phosphorylation sites (Scruggs et al. 2009). There is evidence that MyBP-C binds to the myosin S2 region and RLC (Kampourakis and Irving 2015; Ratti et al. 2011). This indicates that cRLC phosphorylation events are linked to phosphorylation of other sarcomeric proteins. Signaling pathways may not control the phosphorylation of single proteins: the physiological response to stimuli may be a balanced increase in phosphorylation of a number of target sites.

In the heart, the overall pattern of contraction depends on a spatial gradient of RLC phosphorylation that is closely related to cardiac torsion (Davis et al. 2002), the consequence of which is an increased ejection fraction and cardiac stretch activation response (Davis et al. 2001). It was proposed that this variation of RLC phosphorylation ranging from high levels in the epicardium to low levels in the endocardium could be due to phosphatase activity that may be higher in the inner layers of the myocardium. This, in turn, could possibly be a mechanism to reduce inner wall stress of the heart during systole (Cohen 2002; Rajashree et al. 2005). These studies suggest that the gradient of RLC phosphorylation supports the cardiac torsional movement and thus facilitates overall cardiac contraction. Interestingly, a recent study in rat heart shows that an increase in RLC phosphorylation slows down the rates of both cross-bridge MgADP release and MgATP binding at 1.9-µm sarcomere length, whereas only slows down cross-bridge MgATP binding at 2.2-µm sarcomere length with no effects on the rate of MgADP release (Pulcastro et al. 2016). This indicates that RLC phosphorylation has a differential role on cross-bridge kinetics depending on sarcomere length. This connects to and adds to the concept that RLC phosphorylation can vary across the heart from left to right ventricles and between fibers based on the local extent of mechanical loading and stretching to optimize normal cardiac torsional movement and overall contraction.

Recently, cardiac RLC phosphorylation was found to directly control the movement of the myosin-RLC region and myosin S1-region from a position more parallel and towards the thick filament backbone to a position more perpendicular and away from the thick filament backbone, facilitating cross-bridge formation, enhancing myosin kinetics, and force production (Kampourakis and Irving 2015). This suggests that RLC phosphorylation is a major modulator in releasing the myosin head–thick filament backbone interaction and inducing movement away from the backbone towards the thin filament. This mechanism agrees well with the hypothesis proposed for super-relaxed state (SRX) of myosin head in cardiac muscle (Hooijman et al. 2011) and for skeletal muscle (Stewart et al. 2010). This ordered “blocked” (lying on thick filament) arrangement of the myosin heads across the thick filament surface in SRX is modulated by phosphorylation of RLC and MyBP-C (Kensler et al. 2011; Levine et al. 1996). Supporting this, de-phosphorylation of RLC stabilizes the ordered array of myosin heads bound or lying on the thick filament surface away from thin filament, decreasing cardiac power output during SRX (Dias et al. 2006; Scruggs et al. 2009).

#### Smooth muscle: the role of RLC phosphorylation

The role of RLC and its phosphorylation is better characterized in smooth muscle and non-muscle cells than in striated muscle. RLC phosphorylation triggers smooth muscle contraction, unlike in striated muscle. Phosphorylation of RLC in both smooth muscle and non-muscle myosins modulates the tension and activates the myosin ATPase (Bresnick 1999). The mechanism by which RLC phosphorylation induces the release of the two-head interactions, a feature specific to smooth muscle to activate the myosin motor is not clearly known. According to molecular dynamics (MD) simulation along with electron paramagnetic resonance (EPR) and fluorescence resonance energy transfer (FRET) experiments, the smooth muscle RLC (smRLC) phosphorylation domain (PD) shifts to a more ordered state from a disordered conformation upon phosphorylation at Ser19 (Espinoza-Fonseca et al. 2007). The overall tertiary structure of the RLC and the light chain domain region of the myosin S1 undergo a shift from a closed or ordered state to a more open or disordered state of conformation (Colson et al. 2012; Taylor et al. 2014) upon phosphorylation. This conformational change could facilitate the release of the myosin S1, activating its ATPase activity. In addition, in non-muscle cells as well, RLC phosphorylation is required for normal cell division processes and cytokinesis. Both the phosphorylation sites and the pattern of phosphorylation characterize striated and smooth muscle RLC phosphorylation and highlight the fact that the consequence of RLC phosphorylation is quite different in these two muscle classes.

### Possible mechanism of RLC phosphorylation in striated muscle

The structural and functional role of RLC phosphorylation has also been investigated in genetic models such as *Drosophila*. X-ray diffraction on live flies and active muscle fibers shows that RLC phosphorylation orients the myosin motor domain towards the thin filament target to enhance actin binding compared to non-phosphorylated RLC (Farman et al. 2009). Cross-bridges without RLC phosphorylation are more ordered in relaxed mammalian skeletal muscle since they lie closer to the thick filament core (Cooke 2011). However, during contraction, skeletal muscle cross-bridges with phosphorylated RLC rotate at higher azimuthal angles and are less ordered compared to those without RLC phosphorylation (Midde et al. 2013). This is in contrast to what is found in insect flight muscle (Farman et al. 2009) where RLC phosphorylation reduces the azimuthal movement of the myosin head, enabling a better orientation towards actin targets. Moreover, in active isometric condition, Midde et al. (2013) found that rotational movement of the myosin lever arm (myosin S1’s RLC region) is faster with phosphorylated RLC than with dephosphorylated RLC even with the myosin duty cycle and isometric tension remaining the same in both cases. Upon RLC phosphorylation, the myosin S1 head domain and the S1 RLC region are more disordered compared to non-phosphorylated RLC. However, Duggal et al. reported that in skeletal muscle, RLC phosphorylation has a minimal effect on the ordering of the myosin S1 head domains or their distribution (Duggal et al. 2014). These contrasting results highlight the need for a better understanding of the role of RLC phosphorylation and that RLC phosphorylation may have different consequence in different tissues or animal systems. In summary, RLC phosphorylation causes disordering of the myosin head domain but not the RLC-region of S1. The S1 RLC region may remain ordered due to its interaction with the thick filament backbone. Phosphorylation may shift this structural equilibrium possibly due to the electrostatic repulsion of phosphate with the negatively charged surface of the thick filament backbone. Alternatively, whether the effect of RLC phosphorylation is to disorder the S1’s RLC-region and S1’s myosin head domain or to just shift the equilibrium of the interaction between S1’s RLC region and thick filament backbone surface to an “ON” state (not referring to calcium activation) may depend on the type of muscle skeletal (Midde et al. 2013), or cardiac (Kampourakis and Irving 2015). In the ON-state, it is proposed that due to RLC phosphorylation, the myosin head and S1-RLC region axis is more perpendicular to the thick filament backbone, whereas without phosphorylation in the OFF-state, it is more parallel to the thick filament axis (Kampourakis and Irving 2015) (see Fig. 3). RLC phosphorylation releases the inhibition (OFF state) caused by interaction between myosin S1 head–head and S1’s head domain/S1 RLC region with the thick filament backbone interaction to facilitate head and lever arm movement away from the backbone and towards the thin filament, based on evidence from smooth muscle (Baumann et al. 2012; Rosenfeld et al. 1998), permeabilized mammalian cardiac muscle studies (Colson et al. 2010), and *Drosophila* indirect flight muscle (Farman et al. 2009). A summary of the known physiological functions of RLC phosphorylation in striated muscle is shown in Table 1.

**Table 1:** Known physiological effects of RLC upon phosphorylation

	Skeletal RLC	Cardiac RLC	References
Associated kinases	skMLCK, ZIP kinase, PKC, etc.	cMLCK, ZIP kinase, PKC, etc.	Allen et al. (2008), Chan et al. (2008), Chang et al. (2015), Scruggs and Solaro (2011), Stull et al. (1986)
Ca^2+^ sensitivity	Increases	Increases	Olsson et al. (2004), Szczesna et al. (2002)
Ca^2+^-dependent force	Increases	Increases	Olsson et al. (2004), Sweeney and Stull (1990), Toepfer et al. (2013)
Maximum Ca-activated force	Increases	Increases	Olsson et al. (2004), Sweeney and Stull (1990), Szczesna et al. (2002), Toepfer et al. (2013)
Rate of force redevelopment/stretch activation	Unknown	Increases	Olsson et al. (2004), Stelzer et al. (2006), Szczesna et al. (2002)
Myosin ATPase activity/cross-bridge cycling	Increases	Increases	Colson et al. (2010), Olsson et al. (2004), Sweeney and Stull (1990), Szczesna et al. (2002), Toepfer et al. (2013)
Role in cross-bridge	Induces movement of myosin head away from thick filament backbone towards actin; increased disorder	May induce movement of myosin head and RLC-region away from thick filament backbone towards actin; not increasing disorder	Duggal et al. (2014), Irving et al. (2000), Midde et al. (2013), Ushakov et al. (2011)
Sarcomeric development	Unknown	Induces formation of well-organized sarcomeres; but there is a contrary report showing normal sarcomere pattern and cardiac performance in cRLC without increased phosphorylation	Aoki et al. (2000), Chang et al. (2013)
Phos level under pathological conditions	No change in muscular dystrophic model mouse (*mdx* mouse); not well understood	Decreases in FHC; increases with severity of heart failure	Smith et al. (2010), Szczesna (2003), Szczesna et al. (2001, 2002), Toepfer et al. (2013)
Phos with disease severity	Unknown	Increases	Toepfer et al. (2013)

## Biomedical significance of RLC phosphorylation

As RLC phosphorylation modulates muscle cell behavior, defects in RLC phosphorylation result in dysfunction and disease. Mutations in either ELC or RLC are associated with myopathies in human heart and skeletal muscle (Poetter et al. 1996).

In skeletal muscle, RLC phosphorylation plays a role in post-activation potentiation, which is induced by a sudden and intense voluntary contractile activity like jumping or sprinting etc. (Baudry and Duchateau 2007; Sale 2002). The role of RLC phosphorylation in post-activation potentiation may stimulate research to improve human performance in stretch (extrinsic) and endurance exercise. RLC phosphorylation and increased recruitment of motor units have been proposed as the mechanism of post-activation potentiation that leads to increased peak force and rate of force development during twitch contractions. Increasing RLC phosphorylation may be an effective strategy for improving skeletal muscle performance in athletes as well as in the aging population.

In the heart, genetic knock-out mice were used to understand the role of the ventricular and atrial isoforms of RLC, MYL2v, and MYL2a, respectively, in cardiac contractile function (Sheikh et al. 2015). The mutations of RLC are associated with the mid left ventricular chamber hypertrophic cardiomyopathy (HCM) phenotype. Although it is not entirely clear how these abnormal phenotypes are driven by changes at the molecular level, RLC mutants linked to these diseases are known to modify the cardiac myosin lever arm (Hernandez et al. 2007). Burghardt et al. studied the relationship between different disease-related mutants in RLC and heart diseases using photo-activatable GFP-tagged RLC (RLC-PAGFP), a novel technique that permits single-molecule detection (Burghardt and Sikkink 2013). They found that the HCM-linked E134A mutation of RLC, a charged residue probably participating in the binding of human cardiac RLC to the myosin lever arm, affects actin binding during contraction evidenced by a decrease in isometric tension.

Cardiac hypertrophy is the heart’s response to a variety of extrinsic and intrinsic stimuli that impose increased biomechanical stress (Frey and Olson 2003). Increased cardiac RLC phosphorylation may limit cardiac hypertrophy by contributing to enhancement of contractile performance and efficiency. This may result from facilitating actomyosin interactions and enhancing contractility to adapt to cardiac stress (Huang et al. 2008; Muthu et al. 2012). Additionally, pressure overload leads to severe heart failure in mice with dephosphorylated cardiac RLC but less so in mice with over-phosphorylated cardiac RLC (Warren et al. 2012). Thus, strategies to control RLC phosphorylation may benefit heart function in disease.

In addition, measurement of the extent of RLC phosphorylation has potential as a diagnostic tool for human heart disease phenotypes. A recent study tested cardiac left ventricular (LV) biopsy samples from patients either with LV hypertrophy and preserved systolic function or with LV dilation and reduced ejection fraction (Walker et al. 2013). In patients with preserved systolic function, cardiac RLC phosphorylation was unchanged relative to a control group of patients with normal LV function, whereas in patients with LV dilation and reduced ejection fraction, cardiac RLC phosphorylation was increased. A role for phosphorylation of RLC in various cellular functions and diseases is evident. Thus, understanding the stage-specific changes in phosphorylation of RLC in progressive heart diseases, either heart failure or cardiomyopathic conditions, may provide important and early diagnostic and therapeutic information.

In the paragraphs that follow, we will present a summary of existing methods for measuring and modifying the extent of RLC phosphorylation and discuss their merits and problems.

## Techniques to phosphorylate or dephosphorylate RLC

Techniques to phosphorylate or dephosphorylate RLC include computational simulation, modeling, and experimental methods. Mathematical simulations describe the phosphorylation and de-phosphorylation dynamics of RLC by modifying biochemical and enzymatic parameters (Kaneko-Kawano et al. 2012). However, simulations need validating by experimental data. Here, we emphasize experimental techniques for: (1) increasing phosphorylation of RLC, and (2) techniques to de-phosphorylate RLC, and (3) the measurement of phosphorylation levels. The merits and limitations of the experimental tools will be discussed, divided into in vivo and in vitro methods.

## In vivo modifications

Much of our knowledge about native myosin light-chain functions comes from studies in muscle of animal models such as mouse, rat, rabbit, swine, zebrafish, and *Drosophila* (Kaneko-Kawano et al. 2012). Several studies have measured the extent of native RLC phosphorylation in various tissues that seems to reflect variation in the phosphorylation extent between them. In quickly frozen biopsy samples of human heart, RLC phosphorylation varies between the atrial and the ventricular isoforms, with the two ventricular isoforms varying in the range of 0.26–0.39 mol Pi/mol RLC (Morano 1999). Toepfer et al. (Toepfer et al. 2013) found a similar level of RLC phosphorylation (~0.4 mol Pi/mol RLC) at the inner ventricular wall of the human myocardium. However, basal RLC phosphorylation values found in different studies differ (Peng et al. 2014; Sanbe et al. 1999; Scruggs and Solaro 2011). Heterogeneous levels of phosphorylation between different parts of the myocardium (Davis et al. 2001; Dias et al. 2006; Sheikh et al. 2012) add to the complication. For example, recently, the basal in vivo cRLC phosphorylation in the rodent heart is found to be even lower (<0.05 mol Pi/mol RLC) (Kampourakis and Irving 2015) than reported before (~0.4 mol Pi/mol RLC) (Toepfer et al. 2013). The difference could also be caused by handling such as rapid dephosphorylation during sample preparation. Previously, in case of rodent models, RLC phosphorylation level in the inner left ventricular wall of rats hearts is found to be higher than in human (0.53 mol Pi/mol RLC) (Toepfer et al. 2013). However, normal RLC phosphorylation levels in swine left ventricular wall is similar to that in humans (Toepfer et al. 2013). In case of skeletal muscle, the extent of normal RLC phosphorylation is lower than in cardiac muscle, with the mouse fast twitch EDL muscles showing about 0.1–0.2 mol Pi/mol RLC, and the mouse slow twitch soleus muscle showing about 0.08–0.18 mol Pi/mol RLC (Ryder et al. 2007). Therefore, in vivo modification of RLC phosphorylation status may result in directly measurable physiological effects.

### Transgenic approaches: modulation by kinases

Transgenic techniques are widely used for in vivo modification, most of which are used to lower the RLC phosphorylation level. For this purpose, modulation of MLCK expression is frequently used. Mammalian smooth, skeletal, and cardiac muscle MLCKs are encoded by *MYLK1*, *MYLK2*, and *MYLK3* genes, respectively (Gallagher et al. 1991; Herring et al. 2000; Yin et al. 2006; Zhi et al. 2005). Ding et al. generated a transgenic mouse expressing the cMLCK hypomorphic allele (*cMLCK* ^*neo/neo*^) by crossing *cMLCK* ^+*/neo*^ to each other in order to assess myosin RLC phosphorylation and cardiac performance in vivo (Ding et al. 2010). Two cMLCK-targeted embryonic stem cell clones were identified and injected into C57BL/6 blastocysts that were transferred to the uterus of pseudo pregnant females. The extent of RLC phosphorylation in ventricular and atrial tissues of *cMLCK* ^+*/neo*^ mice was less than 5 %, indicating successful dephosphorylation by modulating MLCK. In another in vivo study, skMLCK-deficient mice were generated by transferring *MYLK2* knock down into C57BL/B6 blastocysts and implanting into pseudopregnant C57ICR females (Zhi et al. 2005). Loss of skMLCK reduced RLC phosphorylation in skeletal muscle by 95 % but had no effect on cRLC phosphorylation.

It has been shown in smooth muscle that when tamoxifen treatment is combined with transgenic specific promoter targeting and tamoxifen-activated Cre recombinase (a tyrosine recombinase enzyme derived from the P1 Bacteriophage), smMLCK expression is tissue-specifically abolished (Kuhbandner et al. 2000). This resulted in lower levels of RLC phosphorylation and consequent functional implications (He et al. 2008). A similar strategy using tamoxifen activated and tissue-specific knock-out of smMLCK in vivo created a transgenic mouse strain with very low levels of RLC phosphorylation in smooth muscle (Somlyo et al. 2004; Zhang et al. 2010). This transgenic strategy could be applied in striated muscle as well. Thus, targeting MLCK genetically or in combination with pharmacological intervention is effective for tissue-specific modulation of RLC phosphorylation. In vivo modification of MLCK is an effective technique for examining the function of RLC phosphorylation in specific muscle tissue systems.

### Transgenic approaches: P-element-mediated germline transformation

In vivo transgenic approaches were utilized in *Drosophila* to understand the role of the conserved MLCK phosphorylation sites of RLC in the stretch activation response, which has features resembling the behavior of mammalian cardiac muscle (Dickinson et al. 1997; Farman et al. 2009; Miller et al. 2011; Tohtong et al. 1995). Transposon-element or P-element-mediated germ-line transformation was used for creating transgenic *Drosophila* strains expressing mutant RLC with alanine substitutions of MLCK phosphorylatable serines (Ser66 and Ser67 of *Drosophila* RLC: Fig. 2) (Farman et al. 2009). Analysis of the mechanical response of permeabilized *Drosophila* flight muscles to applied sinusoidal length changes suggested that MLCK phosphorylation of the two conserved serines are required for normal orientation of the myosin heads towards actin target zones. This, in turn, accelerated myosin kinetics, increasing the number of strongly bound cross-bridges and stretch-activated power output (Miller et al. 2011).

### Transgenic approaches: pseudo-phosphorylation

Pseudo-phosphorylation is used both in vitro and in vivo. In this method, one or more amino acid residues are mutated to an acidic amino acid [aspartic acid (D) or glutamic acid (E)], which carries a negative charge that mimics the charged state of a phosphorylated residue. Based on the known cRLC crystal structure and on molecular dynamics (MD) simulations, the in vivo phosphorylatable site of Ser15 is proximal to the D166V (Aspartate166 → Valine) mutation site of cRLC known to cause familial hypertrophic cardiomyopathy (FHC). Muthu et al. created a S15D (phosphorylated Ser15) pseudophosphorylated mutation site in the cRLC protein carrying the D166V mutation (Muthu et al. 2014). They found that this in vitro strategy rescues the charged state of the aspartic acid (D166) site and the contractile function in vitro since the Ser15 and D166 sites are proximal. Recent in vivo research engineered pseudophosphorylated RLC mice by using S15D-D166V mutation technique (Yuan et al. 2015) and found that pseudophosphorylated RLC in the hearts of HCM mice is sufficient to prevent the development of the pathological HCM phenotype. This method can also be used in other types of muscle. A similar strategy of combining MD simulations and pseudophosphorylation has also been utilized in smooth muscle RLC to understand the structural effect of the physiological phosphorylation of Ser19 and the neighboring Thr18 (see Fig. 2) of the smRLC (Espinoza-Fonseca et al. 2008). Therefore, combining in silico MD simulations and pseudophosphorylation is a powerful technique for understanding basic structure–function relationship and also the mechanistic basis of disease-causing mutations of RLC. Pseudophosphorylation not only improves systolic function but also avoids adverse effects resulting from the D166V RLC mutation (Granzier and de Tombe 2015). In such pseudophosphorylation, the phospho-mimic negative charge is constitutive, which means that in vivo, the heart cannot control the charged-state of RLC and dynamically respond to altered demands.

Recently, it was found that in the beating heart, cRLC is constitutively and stably phosphorylated at a level of 0.45 mol Pi/mol RLC due to a balance between cMLCK activity and MLCP (myosin light-chain phosphatase) activity (Chang et al. 2015). Thus, pseudophosphorylated RLC along with unphosphorylated RLC at in vivo ratios can be used for exchange into muscle fibers or myofibrils to test functional implication of RLC phosphorylation in normal and disease conditions, assuming that the D substitution is physiologically analogous to serine phosphorylation in vivo. The drawback of this S15D mutation approach is that it will not easily be adapted to patients. A possible alternative is the manipulation of the activities of either cMLCK (increasing it) or cMLCP (lowering it) (Granzier and de Tombe 2015). The reason is that these enzymes are highly specific toward cRLC, and therefore the technique may be feasible without adversely affecting effects on other proteins (Kamm and Stull 2011).

### Direct phosphorylation

In addition to transgenic methods, RLC can be directly phosphorylated in vivo. For example, the hypertrophic agonist angiotensin II (AngII) plays an important role in regulating vascular tone as well as in maintaining systemic blood pressure. Volatile anesthetics may inhibit AngII-induced vascular contraction via inhibiting RLC phosphorylation (Qi et al. 2009). Aoki et al. injected AngII and phenylephrine into the jugular vein of adult rats to promote phosphorylation of cRLC in the heart (Aoki et al. 2000). AngII and phenylephrine increased the basal level of phosphorylated RLC by 30 to 38 % and by 45 %, respectively. The increased RLC phosphorylation is caused by activation of the Ca^2+^/calmodulin pathway activation and subsequent MLCK activation. This method induces the formation of well-organized sarcomeres in the cardiomyocytes. In other studies, mice were treated with β-adrenergic agonists like Isoproterenol (Isoprenaline), which induces cardiac hypertrophy in wild-type mice and is associated with an increase in MYL2v phosphorylation in mice that lack cMLCK, by activating the ZIP Kinase (Ding et al. 2010). A recent study infused AngII type 1 receptor (AT1R) biased ligand, namely TRV120023, into DCM mice for 15 min. The observed enhancement of cardiac contractility was associated with an increase in ventricular MYL2 phosphorylation activated by the β-arrestin downstream signaling pathway (Tarigopula et al. 2015). AT1R may prove to be a novel inotropic approach in familial DCM. Other inotropic agents such as pimobendan and levosimendan probably inhibit phosphodiesterase, which may limit their use in DCM (Kass and Solaro 2006; Nonaka et al. 2015). These studies suggest that hypertrophic stimuli act upstream of cRLC phosphorylation. These pharmacological stimuli can be used to understand the function of cRLC phosphorylation in disease processes. The phosphorylation level achieved exceeds the physiological levels and thus may not reflect an in vivo mechanism. However, lowering doses of hypertrophic agonists may achieve physiological levels of RLC phosphorylation. Agonist administration is invasive, so side effects need to be checked, such as compensatory over- or under-expression of other proteins and non-physiological phosphorylation of RLC or other proteins. RLC phosphorylation is manipulated directly with agonists but the effects are difficult to control. Additionally, a study used 15-Hz tetanic stimulation in skMLCK knock-out mice, and found some RLC monophosphorylation (Zhi et al. 2005). However, it may be caused by redundancy between skMLCK and smMLCK, so this method is not widely used (Takashima 2009a).

Nevertheless, in vivo phosphorylation targets specific RLC isoforms, which allows exploration of phosphorylation effects in different muscle types under physiological conditions. Commonly used in vivo methods are summarized in Table 2.

**Table 2:** Comparison of commonly used in vivo RLC phosphorylation methods

Techniques	Examples	Phosphorylation efficiency	Advantages	Disadvantages	References
Transgenic	Manipulate MLCK expression by cloning/RNAi, P-element-mediated germline transformation (*Drosophila*), in vivo pseudo-phosphorylation	Decreased by 95 % or significantly increased, constitutive phosphor-mimicking state in pseudophosphorylation	Able to modify specific gene/site of interest in physiological setting; Able to investigate the effect of phosphorylation from the molecular to the whole-organism levels	Expensive, time-consuming, and creating a transgenic model is generally risky; side effects; false positives; positional effects; not feasible to be performed in patients	Ding et al. (2010), Espinoza-Fonseca et al. (2008), Farman et al. (2009), Miller et al. (2011), Muthu et al. (2014)
Direct phosphorylation	Injection of angiotensin II/AT1R/phenylephrine/isoproterenol	Increased by 30–45 %	Physiological conditions; Easy to perform; Cost-effective	The proteins and sites on the proteins of which phosphorylation is being manipulated is not easy to control and may not be physiological in nature; The dosage needs optimization	Aoki et al. (2000), Ding et al. (2010), Huang et al. (2008), Verduyn et al. (2007)

## In vitro modification

In vitro modification of RLC phosphorylation levels is relatively simple and gives high phosphorylation efficiency. In vitro methods include enzymatic, genetic, chemical, biological approaches, and direct physico-chemical methods.

### Overview of kinase methods

Kinases used for in vitro modification of the phosphorylation state include MLCK, PAK, and Rho-kinase. The MLCK family is a set of calmodulin-dependent protein kinases which readily phosphorylate different RLCs. Smooth muscle MLCK (smMLCK) is expressed in most tissue including smooth muscle, skeletal muscle, and cardiac muscle. smMLCK catalyzes Ca^2+^/calmodulin-dependent phosphorylation of RLC in epithelial, endothelial, and other kinds of non-muscle cells. The smMLCK is sufficient for initiating smooth muscle contraction or actomyosin-dependent movement in non-muscle cells (Yu et al. 2010). Thus, the smooth muscle MLCK expressed by the *MYLK* gene is ubiquitous. In smooth muscle cells, the concentration of smMLCK and its substrate, RLC, is about 4 μmol/l and 30–40 μmol/l, respectively (Takashima 2009b). The skMLCK is a distinct kinase mainly found in skeletal muscle, particularly in fast-twitch fibers (Allen et al. 2008). Although it was reported in cardiac muscle, the amount is too low to maintain cardiac RLC phosphorylation (Davis et al. 2001) as demonstrated by knock-outs of skMLCK with no effect on cRLC phosphorylation (Zhi et al. 2005). The skMLCK is not selective, as it phosphorylates RLCs from smooth, skeletal, and cardiac muscles with similar maximal rate (*V*_max_) and Michaelis constant (*K*_m_) (Stull et al. 1986). Although skMLCK is activated by Ca^2+^ and the catalysis core recognizes the RLC binding site, the mechanism is different from that of smMLCK. Under steady-state conditions, skMLCK follows a rapid-equilibrium random bi–bi reaction (two substrates converted to two products) whereas smMLCK proceeds by an ordered sequential mechanism in which Mg-ATP binds first, followed by the RLC substrate (Bowman et al. 1992). cMLCK is a kinase first found in 2008 and only present in vertebrate cardiac ventricular and atrial myocytes (Chan et al. 2008). The cMLCK has a catalytic core domain and regulatory segment that are conserved in skMLCK. However, there is uncertainty about whether the cMLCK kinase ability to phosphorylate RLC is Ca^2+^/calmodulin dependent or not. In rat cardiomyocytes, cMLCK phosphorylates RLC without Ca^2+^/calmodulin dependence in vitro (Chan et al. 2008). However, at around the same time, human cMLCK was found to be Ca^2+^/calmodulin dependent in vitro (Seguchi et al. 2007). Recently though, it has been shown in rat trabeculae that cMLCK indeed phosphorylates cRLC in a Ca^2+^/Calmodulin-dependent manner both in vitro and in situ (Kampourakis and Irving 2015). The phosphorylation efficiency was about 100 % (1 mol Pi/mol RLC) and the fact that in vivo multiple phosphorylated species of cRLC are present may be explained by the presence of different kinases for cRLC other than cMLCK in rodent heart (Chang et al. 2010; Jiang et al. 2014; Venema et al. 1993).

MLCKs have been used to phosphorylate RLC in muscles from various species such as rabbit, mouse, rat, and *Drosophila* (Stull et al. 2011). MLCK is available commercially. Alternatively, it can be recombinantly isolated and purified in the laboratory (Greenberg et al. 2009). Besides MLCK, other kinases such PAK are also widely used in RLC phosphorylation. A detail review will be given below.

### Phosphorylation and de-phosphorylation methodology targeting or using kinases

In vitro phosphorylation of cRLC is simply achieved by incubating 0.5 µM of MLCK with zipper-interacting kinase (ZIP kinase) dissolved in buffer (containing CaCl_2_, MgCl_2_, ATP, etc.) and incubated with 0.1–0.5 µM RLC for 0.5–2 h at 25–37 °C or at 4 °C overnight. This method yields almost 100 % cRLC phosphorylation, i.e., the cRLC species are phosphorylated at Ser15 position with almost 100 % efficiency (Toepfer et al. 2013). A higher concentration of MLCK may result in diphosphorylation. The ZIP Kinase is added along with cMLCK in order to increase RLC phosphorylation to the required level, since ZIP Kinase directly phosphorylates cRLC independently of MLCK pathway (Chang et al. 2015).

Using purified cMLCK catalytic domain along with Ca^2+^/calmodulin, ATPγS thiophosphorylates Ser15 of cRLC that was labeled by bifunctional sulforhodamine (BSR). ATPγS was used because thiophosphorylated proteins are relatively resistant to dephosphorylation by contaminating or endogenous protein phosphatases in the muscle tissue samples (Gratecos and Fischer 1974). In this study (Kampourakis and Irving 2015), at a cMLCK:cRLC ratio of 1:500 or 1:200, almost 100 % of cRLC was mono-phosphorylated. Thiophosphorylated cRLC was then exchanged into demembranated muscle fibers to visualize the orientation of myosin S1’s cRLC region in the acto-myosin machinery using polarized fluorescence measurements in resting, active, and rigor conditions. Although, only about 10–30 % exchange of the BSR-labeled thiophosphorylated cRLC was achieved, this may closely mimic the basal in vivo level of phosphorylated cRLC (40–50 %) in the contracting myocardium as was reported before (Herring and England 1986; Silver et al. 1986). Exchange and incorporation of BSR-labeled thiophosphorylated cRLC had minimal effect in overall protein composition of the permeabilized tissue, only lowering the total amount of few proteins. Significantly though, exchange of the BSR-thiophosphorylated cRLC caused lowering of the amount of phosphorylated TnI and phosphorylated MyBP-C.

Recombinantly expressed RLC is dephosphorylated with shrimp alkaline phosphatase in vitro (Toepfer et al. 2013). MLCK inhibitors such as 50 µM of ML-9 and 400 µM of nonapeptide rkkykyrrk-NH_2_ (D-PIK) are also used to reduce RLC phosphorylation in vitro (Feighery et al. 2008). Mixing specific ratios of phosphorylated and de-phosphorylated RLC prior to light-chain exchange into permeabilized cells effectively provides control of the phosphorylation. For example, in vivo conditions of RLC phosphorylation without tampering with the phosphorylation level of other sarcomeric proteins are achieved. The approach takes advantage of the fact that RLC is readily exchangeable in permeabilized muscle cells (Borejdo et al. 2001; Caorsi et al. 2011; Toepfer et al. 2013). RLC dissociates spontaneously from the myosin heavy chain and diffuses out of the filament lattice (Yang and Sweeney 1995). Addition of RLC to the bathing medium of RLC-extracted permeabilized muscle cells restores functional activity as measured by calcium-dependent force generation and relaxation and velocity-dependent force generation, indicating that RLC effectively re-binds to its myosin target. RLC extraction of up to 80 % can be achieved, accompanied by some extraction of myosin-binding protein C and of troponin components, principally TnC (Toepfer et al. 2013). Inclusion of troponin and MyBP-C in the incubation medium improves the recovery of physiological function as these proteins also reversibly dissociate from the myofilament during RLC exchange and are replenished from the incubation medium. Moreover, as shown recently (Kampourakis and Irving 2015), either chemical permeabilization procedures using detergents (Triton X-100) or treatment of the tissue with solutions containing EGTA (ethylene glycol tetraacetic acid) or BDM (2,3-butanedione monoxime) have no effect on the RLC phosphorylation level. The RLC exchange procedure in chemically permeabilized or demembranated muscle fibers or myofibrils is therefore convenient, and provides precise control of phosphorylation levels of RLC incorporated into the filament lattice. This avoids complications resulting from inadvertent changes in the phosphorylation levels of other sarcomeric proteins, as would be the case if the kinases or phosphatases are added directly to the permeabilized cells. This method also has the advantage that RLC carrying mutations or RLC that have been engineered to carry fluorescent reporters at specific sites can readily be introduced into the muscle fibres or myofibrils. However, in vitro phosphorylation may not mimic the in vivo MLCK activity as the kinases may target different sites depending on whether phosphorylation occurs in vivo or in vitro.

Reduction of phosphorylation is achieved using selective apoptotic agents targeting MLCK, as has been shown in smooth muscle cells (Fazal et al. 2005). Moreover, MLCK inhibitors such as 50 μM of ML-9 and 400 μM of nonapeptide rkkykyrrk-NH2 (D-PIK) are used to reduce RLC phosphorylation in vitro (Feighery et al. 2008). Another method for reducing RLC phosphorylation is by transfecting cultured cells with anti-sense MLCK, as used by Petrache et al. who found that caspase-dependent cleavage of MLCK was implicated in pulmonary endothelial cell apoptosis (Petrache et al. 2003). Caspase-mediated cleavage of MLCK as used in this study could potentially be used as another tool for inhibiting MLCK and reducing RLC phosphorylation in striated muscle in vitro. Inhibitory antibodies against MLCK to block RLC phosphorylation are used, but the effectiveness and reliability of this method depend on the specificity of the antibodies (Fazal et al. 2005).

Other commonly used kinases for RLC phosphorylation are Rho-associated kinase (Rho-kinase) and protein kinase C (PKC). Rho-kinase is also involved in RLC phosphorylation in striated muscle. For example, adult rat ventricular myocytes were treated with the Rho-kinase inhibitor Y-27632 at a concentration of 10 µM so that it does not phosphorylate the myosin-binding phosphatase targeting subunit (MYPT), the catalytic subunit of protein phosphatase 1 (PP1) (Rajashree et al. 2005). This results in inhibition of RLC phosphorylation. It used an antibody (12896, Santa Cruz) to target phosphor-Thr18 and Ser19, which are phosphorylation sites of smooth muscle and non-muscle cells. It should be verified by antibody that targets phosphor-Ser 15. However, this study still suggests an important role for α-adrenergic signaling in modulation of RLC phosphorylation via inhibition of the myosin phosphatase.

The phosphorylation pattern of cRLC by PKC is different from that by MLCK (Scruggs and Solaro 2011) (Venema et al. 1993). In adult rat heart myofibrils in vitro, PKC mediated a phosphorylation of RLC with a stoichiometry (0.7 mol of phosphate/mol of protein) similar to that mediated by MLCK (Venema et al. 1993).

Other kinases such as p21-activated kinase (PAK) and P34 kinase also affect RLC phosphorylation, but most reports refer to smooth muscle and non-muscle cells (Chu et al. 2013).

For example, γ-PAK, that is activated by GTP-binding proteins Cdc42 and Rac, catalyses phosphorylation of intact non-muscle myosin-II and isolated recombinant RLC (Chu et al. 2013). However, the PAK isoform of smooth muscle tissue, known as PAK1, affects RLC phosphorylation differently. By incubating guinea pig Triton-permeabilized smooth muscle tissue with active PAK1, it was found that PAK1 phosphorylates and inhibits smMLCK which reduces RLC phosphorylation. This, in turn, reduced the rate of smooth muscle contraction (Wirth et al. 2003). Purified P34 kinase (cyclin-p34cdc2) also phosphorylates RLC of cytoplasmic and smooth muscle myosin-II in vitro on Ser1 or Ser2 and Thr9 (Satterwhite et al. 1992). The procedures for using these kinases are similar to that for MLCK: incubation of the kinase with myosin RLC for 30 min at 4, 25, or 37 °C (depending on the type of RLC) achieves a high level of phosphorylation (Satterwhite et al. 1992).

These in vitro smooth muscle strategies using various kinases can be applied to striated muscle by using the engineered recombinant RLC and exchanged into muscle fibers or myofibrils.

### Genetic engineering and pseudophosphorylation

Genetic engineering is used to explore RLC expression and phosphorylation (Koga and Ikebe 2008; Lossie et al. 2012). Using RNA interference (RNAi) to interfere Rho1 (Rho-kinase) expression in *Drosophila* S2 cells, the role of Rho-kinase-dependent RLC phosphorylation in the localization of myosin was examined (Dean and Spudich 2006). The use of RNAi is powerful and versatile. Whole genome-wide maps of promoter and enhancer sequences for all genes open the door to targeted and controlled genetic expression to study their effects and functions with the caveat of potential secondary over- or under-expression effects on other genes in the cell due to false targets stemming from similarity in gene sequences. The technique could be utilized in cultured striated myocytes.

Pseudophosphorylation of RLC is another genetic approach to modulate phosphorylation. This technique is the same as discussed above, but is also used in vitro to engineer recombinant RLC. Pseudophosphorylated RLC is studied by in vitro biochemical techniques or is exchanged into muscle fibers (Muthu et al. 2014). (See above “In vivo modifications” section for details).

### Chemical and biological factors

Chemical and biological factors inhibit myosin light chain phosphatase or activate phosphorylation of RLC via signaling pathways. Yamakita performed in situ phosphorylation of smooth muscle and non-muscle RLC in dividing REF-4A cells using [^32^p] orthophosphoric acid (Yamakita et al. 1994). At a concentration of 1 U/ml, thrombin, which is a serine protease, induces a prolonged increase in the phosphorylation of RLC, reaching a peak after 1 min of exposure to thrombin and maintaining elevated phosphorylation for at least 30 min (Amerongen et al. 1998; Richardson et al. 2000). These strategies can also be used in striated muscle cells to modulate RLC phosphorylation levels. For instance, myofibrils from rabbit psoas muscle are dephosphorylated completely by incubation in glycerinating solution containing 4 mM ATP incubating for 60 min at 24 °C. Subsequently, phosphorylated muscle is prepared by inhibiting the activity of phosphatase with the addition of 20 mM NaF and in the presence of 20 mM phosphate along with 5 mM ATP (Stewart et al. 2010).

### Chemical genetic approach

Genetic or a pharmacological approaches alone (discussed above) have several disadvantages, especially when targeting a specific kinase to modulate RLC or other sarcomeric protein phosphorylation. Since the ATP-binding sites of kinases are highly conserved, it is difficult to target kinases specifically using pharmacological inhibitors. On the other hand, genetic approaches to deactivate kinases are very specific but take time to be effective and levels of inactivation are difficult to control. Small molecule pharmacological inhibitors inactivate kinases rapidly and in a dose-dependent manner. Moreover, interpretation of results using genetic or pharmacological techniques is complicated by functional redundancy of kinases and the overlapping substrates of multiple kinases. To overcome this, Kevan Shokat and his colleagues (Garske et al. 2011) developed a chemical genetic approach. A mutation is created to change a conserved hydrophobic amino acid acting as a gatekeeper in the active site of kinases to a small amino acid such as alanine or glycine to generate a uniquely targetable mutant kinase. This mutated kinase can then be targeted with chemically bulky analogs of natural kinase inhibitors to occupy the new enlarged pocket of the gatekeeper site, whereas wild-type kinases will not be inhibited. The effectiveness of the method depends on a mutation that retains kinase activity, which is not achieved in ~30 % of cases. Moreover, non-covalent inhibitor binding limits the power of this method. An alternate method which does not involve bulky amino acid substitution is the engineering of a cysteine gatekeeper in the active site that can be inhibited with an electrophilic inhibitor. The advantage of a cysteine gatekeeper is that it is rare in the kinome and is inherently hydrophobic in nature as well. This strategy achieves specificity via shape complementarity.

This chemical genetic approach has been exploited to identify RLC as a direct substrate for phosphorylation by ZIP kinase to regulate smooth muscle contraction (Moffat et al. 2011). A ZIP-L93G mutant kinase was created that is capable of utilizing a bulky analog of ATP (P^32^ labeled 6-phenyl-ATP) as substrate. Using SDS-PAGE and autoradiography, RLC was found to be the direct substrate of ZIP kinase in situ. Thus, this approach manipulates RLC phosphorylation in different tissue systems by selectively inhibiting kinases.

### Physico-chemical methods

Physico-chemical methods involve the use of physical perturbation such as microinjection, stretch, and protein exchange to incorporate proteins or factors in vitro to modulate RLC phosphorylation. These methods are used in striated muscle and non-muscle cells. Zeng et al. used two physical methods to modify phosphorylation of RLC (Zeng et al. 2000). The first method was microinjection of PAK2 into endothelial cells which increased the phosphorylated RLC from 0.2 mol PO_4_/mol to 0.37 mol PO_4_/mol RLC in 35 min. Moreover, the reaction was specific for mono-phosphorylation of RLC but not for di-phosphorylation. The second method was by constitutively delivering active PAK2 into bovine pulmonary artery endothelial cytosol by using the osmotic delivery method developed by Okada and Rechsteiner (1982).

Toepfer et al. exchanged native RLC with recombinant RLC phosphorylated in vitro by MLCK and ZIP-kinase into permeabilized trabeculae of the rat heart using the technique developed in Irving and Goldman laboratories (Colson et al. 2010; Toepfer et al. 2013). Trabecular muscle was incubated in three sequential 15-min incubations at 20 °C with fresh 0.5 mg/ml RLC exchange solution incorporating 0.5 mg/ml TnC, to reintroduce TnC that is lost during exchange so as not to alter contractile characteristics of the preparations (Borejdo et al. 2001). Temperature is important for the exchange method because higher temperature causes higher tension, which may damage the muscle. Albeit, in Toepfer et al., exchange was done in the presence of 5 mM MgATP and without calcium (relaxing condition), thus technically no active tension was developed in the muscle fiber. This technique increases the RLC phosphorylation level by about 40 % at 20 °C. The physical exchange method benefits from the ability to introduce fluorescent dyes at specific sites in the recombinant RLC at the same time. The fluorescence signal is helpful to track the extent of exchange. The exchange method can also be applied to ELC (Ushakov et al. 2011), but suitable, reversible conditions are more difficult to establish.

Stretch stimuli on muscle cells have been shown to modulate RLC phosphorylation levels in smooth muscle. In the canine basilar artery, stretch increases smooth muscle RLC tri-phosphorylation at MLCK and PKC sites (Nakayama et al. 2003) indicating that stretch could regulate kinase activity. PKCα and PP2A (Protein phosphatase 2A) have physical and functional connection in smooth muscle cells (Azzi et al. 1998). A muscle pre-load stimulus achieved by stretching rabbit ventricular myocardium under physiological condition increases phosphorylation of RLC and other important sarcomeric components including tropomyosin and TnI (Monasky et al. 2010). This suggests that stretching intact muscle fibers modulates RLC phosphorylation physiologically. However, stretching alone is not specific and can cause biochemical alteration to various proteins in the sarcomere, not just RLC.

Applying isotropic compressive stress or shrinkage using a silicone chamber on murine C2C12 skeletal myoblast cells dephosphorylates RLC via. RhoA phosphorylation by adenylyl cyclase/PKA (protein kinase A) pathway (Takemoto et al. 2015). Studies on adult striated muscle fibers are needed to reveal the role of mechano-sensation induced by stretch or compressive stress on the extent of RLC phosphorylation.

RLC phosphorylation is also correlated with the frequency of stimulation for cardiac muscle contraction (Dias et al. 2006; Silver et al. 1986). These studies reported that increasing stimulation frequency from 0 to 126 beats/min for 30 min produced a frequency-dependent increase in RLC phosphorylation from 0.1 to 0.4 mol Pi/mol RLC in perfused rabbit ventricular septae. Thus, frequency of stimulation modulates RLC phosphorylation in situ.

RLC phosphorylation is also correlated with temperature (Moore et al. 1990). Phosphorylation of RLC decreases with increasing incubation temperature of mammalian skeletal muscle fiber.

Commonly used in vitro RLC phosphorylation methods are compared and summarized in Table 3.

**Table 3:** Comparison of commonly used in vitro RLC phosphorylation methods

Techniques	Examples	Phosphorylation efficiency	Advantages	Disadvantages	References
Directly associated kinases of RLC	Targeting MLCK, Rho-Kinase PKC	Increased by 100–300 %	Easy to perform; low cost	Low specificity of some broad spectrum kinase; variable target sites; Requires condition optimization	Kampourakis and Irving (2015); Kaneko-Kawano et al. (2012); Venema et al. (1993)
Genetic engineering	Engineering Rho or Rac pathway via mutation/RNAi, in vitro pseudo-phosphorylation	Increased by about 58 %, constitutive phosphor-mimicking state in pseudophosphorylation	Specific to RLC; Controllable phosphorylation site	Mainly for non-muscle cells; Imprecise controlled expression; may not mimic in vivo physiological environment	Brzeska et al. (2004); Koga and Ikebe (2008
Chemical/biological	Calyculin A and enzymes such as Thrombin	Increased up to 400 %	Easy to perform; High phosphorylation level	Side effects; non-specific phosphorylation of RLC/other proteins; need dosage optimization	Amerongen et al. (1998); Mills et al. (1998)
Chemical genetic approach	Specifically inhibit or activate only one type of kinase that phosphorylates RLC	100 %	Can be highly specific for a particular kinase; specific to target RLC phosphorylation condition	Some kinases (~30 %) lose function due to bulky amino acid substitution in active site; some ligands might not be very effective in inhibition of kinase due to non-covalent nature of action	Garske et al. (2011); Moffat et al. (2011)
Physico-chemical	Microinjection; osmotic delivery; exchange method; stretch; stimulation frequency	Increased by 40 %	Avoids the side effects; mimic “endogenous” level	Troponin C will be lost during exchange and needs replacing; temperature-dependent efficiency	Dias et al. (2006); Monasky et al. (2010); Silver et al. (1986); Takemoto et al. (2015); Toepfer et al. (2013; Zeng et al. (2000)

## Biomechanical measurements after RLC manipulation

The functional effects of in vitro or in vivo manipulations of RLC phosphorylation need precise evaluation in a physiological setting that mimics the live tissue environment. In an ex vivo setting, measurement of the force response and the ATPase rate of the muscle fiber or the myofibril are needed. Single-filament biophysical experiments such as in vitro motility assays (Karabina et al. 2015), or spin-labeling combined with TIRF microscopy (Burghardt and Sikkink 2013) or laser-trap assays (Previs et al. 2014) provide important information at the molecular level, but single filament or studies of solubilized proteins lack the thick–thin filament sarcomeric lattice structure and suffer from the inability to measure a force response. Moreover, these experiments are not conducive to mimic the contraction cycle of cardiac beating by stretching or releasing per se. Chemically demembranated single muscle fibers or isolated single myofibrils offer ex vivo physiological systems in which RLC is readily exchanged (Borejdo et al. 2001; Caorsi et al. 2011) with controlled levels of phosphorylation. Permeabilized muscle fibers mounted between a force transducer and a piezo-motor or moving coil motor and are exposed to solutions to activate, relax, or put the fibers into rigor. Contraction of muscle fibers is activated by addition of calcium or by temperature-jump (T-jump), or by laser flash photolysis of NPE-caged ATP. The force response is measured directly using the force transducer, whereas actomyosin ATPase of the muscle is measured using a fluorescent phosphate sensor added to the bathing solution that rapidly binds phosphate after ATP breakdown. This provides measurement with millisecond time resolution. Stretch–release protocols explore the effect of force and ATPase before, during, or after stretch of muscle fibers to understand the effect of RLC phosphorylation in cardiac muscle during the heart beat cycle, or when using stretch-release protocols in skeletal muscle as experienced during eccentric exercise such as jumping or sprinting (Bickham et al. 2011; He et al. 2000, 1997; Mansfield et al. 2012; Toepfer et al. 2013). Measurement of force exhibited by single myofibrils is measurable by taking advantage of myofibril adherence to clean glass needles with one of the needle attached to a piezomotor to stretch or release the myofibril. The other microneedle bends under the force produced by the myofibril. The microneedle bending is detected from the moving shadow of the needle, illuminated by a HeNe laser, which is cast onto a double photodiode connected differentially. Thus the needle acts as a force transducer to report force generation by the myofibril. The myofibril is activated and relaxed using calcium-containing or calcium-free solution, respectively, by switching the laminar flow of solution flowing over the myofibril from a double-barreled pipette. The type of solution in which the myofibril is bathed depends on the position of the double-barreled pipette (Song et al. 2013; Tesi et al. 2000, 2002; Vikhorev et al. 2014). Recombinant RLC exchange into myofibril to control phosphorylation and subsequent force responses under different conditions can be measured.

Step changes in length or force and ATPase rate measurements are routinely used to investigate the mechanical transduction process in muscle. Sinusoidal small amplitude length perturbation of the muscle fiber or myofibril over a range of frequencies has also been used to retrieve information on acto-myosin cross-bridge attachment-detachment rates and muscle passive properties (Iorga et al. 2012; Miller et al. 2013). These techniques combined with exchange of RLC with manipulated phosphorylation can further help us understand the role of RLC phosphorylation in muscle contraction.

## Techniques to determine the site(s) and extent of RLC phosphorylation

The measurement of in vivo RLC phosphorylation is still quite a challenge, as evidenced by the reported phosphorylation ratio of skRLC ranging from 5–40 % (Takashima 2009a). Considering the ubiquitous phosphatase activity in tissue, it is important to inhibit phosphatase when preparing sample for phosphorylation analysis. Methods to detect phosphorylation site(s) in normal and diseased tissue samples and for the measurement of the extent of phosphorylation are improving in terms of sensitivity, precision, and throughput. Some of the major approaches in phosphoproteomic analyses are protein mixture fractionation by gel electrophoresis, liquid chromatography, in tandem with phosphopeptide enrichment techniques coupled to mass spectrometry (MS) characterization.

In gel-based approaches, conventional two-dimensional electrophoresis (2DE) gel analysis is most common to detect phosphovariants of proteins according to their isoelectric point by isoelectric focusing and subsequently by molecular weight via SDS-PAGE (O’Farrell 1975). Phosphoproteins and their variants that have been separated by both charge and weight are visualized after staining (Steinberg 2009). For example, autoradiography using ^32^P/^33^P labeling of phosphate groups is possibly the most sensitive, albeit time-consuming, method. Immunoblotting using phospho-specific antibodies is extensively used to detect phospho-Tyr in proteins. The disadvantage is the low abundance of phospho-Tyr species, which makes them hard to detect, and phospho-Ser/Thr antibodies are known to have low specificity. Immunoblotting is performed using antibodies against RLC. Some RLC antibodies are specific for certain RLCs, i.e., cRLC and not smRLC, or specific for the animal type, i.e., human or mouse or rat. However, others are promiscuous, as the amino acid sequence they recognize is conserved across RLC types (Fig. 2).

Fluorescent stains specific for phosphoproteins are used in 1DE and 2DE gels. Pro-Q Diamond (Molecular Probes^®^) selectively stain phosphoproteins in acrylamide gels, without the need for blotting or phosphoprotein-specific antibodies and Western-blot analysis (Schulenberg et al. 2004; Steinberg 2009). Pro-Q stain is not quantitative, but can be used quantitatively if aided by sensitive mass spectrometry. Another technique uses a phosphate-binding tag (Phos-Tag), which binds specifically to phosphorylated ions and thus has a sensitivity to phosphoproteins that is similar to autoradiography. Phos-tag functions at neutral pH for the visualization of phosphorylated proteins in 1DE and 2DE gels (Kinoshita et al. 2006). Using Phos-tag gels, Marston and other groups found different phosphovariants of RLC in cardiac muscle tissues from normal and diseased states (Kinoshita et al. 2009; Toepfer et al. 2013). Moreover, Phos-tag SDS-PAGE can be used to clearly distinguish between thiophosphorylated and phosphorylated RLC as thiophosphorylated RLC migrates faster (Sutherland and Walsh 2012).

The 2DE gel system detects phosphorylation variants and phosphorylation sites (amount and location) as it can be conjugated with protease digestion and tandem mass spectrometry (also known as MS/MS) (Lin et al. 2010). For detection of very low phosphorylation levels in protein samples, phosphorylation enrichment of the sample is used before MS/MS detection. Analyses and detail descriptions can be found in the review by Dunn et al. (2010). Other than gel-based approaches, liquid chromatography along with MS gives sensitive separation and detection of phosphoproteins and phosphorylated sites. Affinity enrichment strategies such as immunoprecipitation and immune-affinity chromatography are used for purification of phosphoproteins followed by peptide fragmentation and MS/MS or tandem mass spectrometry to detect phosphorylation sites. The small molecular weight of RLCs and their acidic nature due to phosphorylation means that the species separate well in 2DE isoelectric focusing (IEF) gels, using tube gel electrophoresis with ampholytes of narrow pH range (4.5–5.4 pH range). The use of tube gels allows a comparatively large amount (~1 mg) of myofilament-enriched homogenate to be loaded, which permits the detection of low levels of phosphorylation or other post-translational modifications by MS or other methods (Westwood and Perry 1981). Analysis of the gel reveals variants based on their phosphorylation states: un-phosphorylated, mono-phosphorylated, and di-phosphorylated. This technique has been used to examine the phosphorylation of RLC in rabbit, rat, and mouse (Frearson et al. 1976).

The caveat of the 2DE system is that it is time consuming. A faster alternative is the 1DE Urea-Glycerol PAGE that has been extensively used for separating RLC species, especially from cardiac muscle tissue samples (Craig et al. 1987; Sobieszek 1986; Szczesna et al. 2002). Adding SDS and performing Urea/SDS-PAGE for cardiac samples has also been used for separating RLC phosphovariants (Kerrick et al. 2009; Muthu et al. 2012). This 1DE gel approach can be combined with western blot analysis by means of antibodies specific to the phosphorylated form of RLC (Davis et al. 2001). Moreover, these procedures are facilitated by selective extraction of RLC from the muscle homogenate before electrophoretic separation as described in the work of Davis et al. (Davis et al. 2001).

## Discussion and perspectives

### In vivo manipulation

Studying the function of RLC phosphorylation in vivo is a major advantage of transgenic techniques, to investigate the effect of phosphorylation from the molecular to the whole organism levels. Transgenic techniques may also allow studies of specific diseases in animal models. However, creating transgenic animals is time-consuming and their maintenance is expensive. A mutation introduced in the live animals may have resulted in the development of secondary unidentified changes which might compensate or accentuate the effects of the mutation, complicating the interpretation of the results. Also, the penetrance of the mutation may be variable and tissue specific (Cooper et al. 2013; Pereira et al. 1994).

Limiting expression levels using RNAi or transgenic targeting techniques may cause false positives because of interactions with multiple genes and isoforms of RLC or MLCK. For example, gene duplication and conversion events of mammalian RLC genes from smooth muscle, and non-muscle cells, lead to a high degree of sequence similarity and consequent difficulties for specific RNAi targeting or transgenesis. With regard to compensatory expression or modification of other proteins in cells carrying mutant constructs in transgenic animals, Scruggs et al. found that transgenically abolishing RLC phosphorylation reduces phosphorylation of both myosin binding protein C (MyBP-C) and TnI in cardiac muscle (Scruggs et al. 2009). These modifications could mask or amplify the effect of the original mutation. Minamisawa et al. demonstrated that a post-transcriptional compensatory program is triggered to maintain normal expression levels and functions even in a heterozygous mouse with a single dosage of ventricular RLC gene (Minamisawa et al. 1999).

Another disadvantage of the transgenic approach is the positional effect of the transgene. Depending on the landing location of the transgene in the genome, expression of other genes along with the transgene can be affected. In spite of these possible side effects, various protein–protein, biochemical, and chemo-mechanical interactions in the muscle and non-muscle tissues are deciphered by in vivo transgenics (Maughan et al. 2005). More recently, advanced technologies such as the phiC31 recombinase systems and the CRISPR/Cas9 system allow precise in vivo mutations or transgenesis in both invertebrates and vertebrates and have the potential to heal by gene therapy (Bischof et al. 2007; High et al. 2014; Imayoshi et al. 2013; Wang et al. 2013). These advances in in vivo genetic technologies will allow more precise in vivo targeting of RLC phosphorylation with regard to specific sites or specific chromosomal locations, or the targeting of specific kinases, which may open the possibility of therapeutic interventions.

In vivo pharmacological treatments are attractive approaches due to their simplicity. However, as Scruggs et al. found, abolishing RLC phosphorylation affects phosphorylation of both MyBP-C and TnI (Scruggs et al. 2009), and general administration of compounds such as AngII (Aoki et al. 2000) may have multiple effects that complicate experimental results and interpretations. In vivo propranolol injection in mice was initially used to dephosphorylate reported TnI in cardiac muscle (Vikhorev et al. 2014). The authors found the TnI phosphorylation was successfully reduced from 1.0 to 0.3 mol Pi/mol TnI and was accompanied with a reduction in the phosphorylation level of MyBP-C from 2.9 to 0.25 mol Pi/mol MyBP-C, but has no effect on RLC phosphorylation (Chang et al. 2015). Thus, since RLC phosphorylation modulation could affect MyBP-C and TnI phosphorylation, propranolol treatment could be used as a negative control to change MyBP-C and TnI phosphorylation without affecting RLC phosphorylation. Although this approach depends on the level of protein dephosphorylation, it may establish the direct physiological and pharmacological effect of RLC phosphorylation on its own.

### In vitro manipulations

Phosphorylation of RLC using an in vitro approach is faster than in vivo for studying mechanisms and for finding therapeutic targets or strategies to ameliorate muscle diseases. Targeting kinases like MLCK, and Rho-kinase, that are directly associated with RLC phosphorylation regulation is effective. The caveat in this approach is the assumption that the kinase acts on physiological in vivo target sites with similar effectiveness as in vitro, which might not be the case. Moreover, some recombinant kinases might have low specificity depending on the tissue origin or if they are bacteriologically expressed. Also, some kinases may have a broad functional spectrum. Therefore, optimization of the in vitro conditions to mimic the physiological environment as much as possible is needed. False positives or negatives might arise in genetic engineering, when using chemical/biological agents such as calyculin A, or when applying chemical genetic approaches or physical methods. Nevertheless, due to their ease and speed, the in vitro techniques have definite advantages over in vivo approaches.

### The molecular players maintaining RLC phosphorylation/dephosphorylation balance

Physiologically RLC phosphorylation in striated or smooth muscle is regulated by the balance of activities between MLCK and the myosin light chain phosphatase (MLCP). There are several molecular players that control this dynamic process. Along with regulation via MLCK activity (reviewed above), MLCP also keeps the activity balance (for detailed review see (Hartshorne et al. 2004). The major functional subunit of MLCP is the myosin phosphatase targeting protein 1 (MYPT1) in smooth muscle, and MYPT2 in skeletal and cardiac muscle. MLCP inhibition is regulated by agonist stimulation via various G protein-coupled receptors and RhoA and Rho-kinase pathways (Hartshorne et al. 1998; Somlyo and Somlyo 2003), mostly targeting the MYPT1 or MYPT2. An additional kinase that phosphorylates and inhibits MLCP is known as MPT1 kinase (Borman et al. 2002). Activation of MLCP is usually related to the increase in the levels of cAMP and cGMP dependent on the activity of protein kinase A/G (PKA and PKG). MLCP activation is known to be fulfilled by PKA/PKG phosphorylation of the MYPT1 in smooth muscle (Nakamura et al. 2007; Somlyo 2007) that opposes the inactivating RhoA/Rho-kinase pathway.

Recently, a novel regulator named myosin phosphatase-Rho interacting protein (M-RIP) was identified in smooth muscle (Gebbink et al. 1997; Surks et al. 2003). M-RIP is a member of the MLCP complex, which associates with the myosin phosphatase targeting protein 1 (MYPT1), the regulatory- and myosin-binding subunit of MLCP. M-RIP directly binds to both RhoA and MLCP at adjacent sites to form the trimer. By binding to actin via the N-terminus, M-RIP links MLCP to the sarcomeric filaments to dephosphorylate RLC (Mulder et al. 2005). However, most of the studies explored smooth muscle cells and non-muscle cells (Riddick et al. 2008; Surks et al. 2005). Besides RNAi, another method described by Mahavadi et al. uses PKG to regulate phosphorylation (Mahavadi et al. 2014). It was found that PKG-induced phosphorylation of M-RIP enhances its association with MYPT1 to increase MLCP activity and RLC dephosphorylation. Thus, M-RIP is a target of PKG to mediate RLC dephosphorylation and smooth muscle relaxation. M-RIP is thus a potential target for modulating RLC phosphorylation in smooth muscle and possibly also in striated muscle.

In striated muscle too, MLCP activity balances the MLCK activity. It has been shown that RLC phosphorylation in fast twitch skeletal muscle fibers is slower than the twitch contraction but is faster than RLC dephosphorylation by MLCP (Sweeney et al. 1993). However, in cardiac muscle the rates of RLC phosphorylation and dephosphorylation are lower than in skeletal muscle, suggesting that this balancing activity is tissue specific and important for modulation of muscle contraction (Sweeney et al. 1993).

### Issues and future directions

The role of striated muscle RLC phosphorylation in health and disease is an emerging question. The quest for understanding the mechanism by which RLC phosphorylation affects muscle function may lead to the development of therapeutic targets. Some of the major issues that need addressing are:

1. How does RLC phosphorylation affect the acto–myosin interactions, the structure of the myosin cross-bridge and the ability of the muscle to generate force?
2. What are the molecular mechanisms by which naturally occurring mutations change RLC phosphorylation levels?
3. How do changes in RLC phosphorylation affect the phosphoproteome of the muscle cells, and how do these changes in phosphoproteome affect cellular behavior and the contractile machinery?
4. Do therapeutic modifications of the RLC phosphorylation level improve contractile performance in disease conditions?
5. What are the in vivo drivers that control RLC phosphorylation? Are these specific to RLC or shared with other proteins? How dynamic are the phosphorylation changes, and on what time scale do they operate?

To answer the above questions, a number of technologies will need to be applied, singly and in combination. Optical and electron microscopy may reveal structural changes brought about by changes in phosphorylation. Low-angle X-ray crystallography and FRET (Förster resonance energy transfer) studies may be used to observe the dynamics of cross-bridge interactions in different phosphorylation states of RLC. These studies may focus on the structure of the cross-bridges, but also in the inter-filament spacing and myofibrillar organization. Pseudophosphorylation may provide stable systems to aid structural studies and molecular dynamic simulations. Results will assist the elaboration of models of contraction in which the contractile performance and efficiency of muscle is related to disease states encountered in hypertrophic or dilated cardiomyopathies.

## Conclusions

RLC phosphorylation regulates muscle contraction and other cellular functions, with important consequences for muscle behavior in health and disease. RLC phosphorylation plays a part in muscle disorders and may be a future therapeutic target. This review summarizes the structure and function of RLC and gives an overview of the methods available for manipulating and testing the effect of RLC phosphorylation in muscle tissue, focusing on striated muscle. We also compare the advantages and disadvantages of in vivo and in vitro methods and suggest their potential applications.
